# Examining the Incidence of Human Papillomavirus-Associated Head and Neck Cancers by Race and Ethnicity in the U.S., 1995–2005

**DOI:** 10.1371/journal.pone.0032657

**Published:** 2012-03-20

**Authors:** Lauren Cole, Linda Polfus, Edward S. Peters

**Affiliations:** 1 Louisiana State University Health Sciences Center, School of Public Health, Epidemiology, New Orleans, Louisiana, United States of America; 2 The University of Texas Health Science Center at Houston, Human Genetics Center, Division of Epidemiology, Human Genetics, and Environmental Sciences, School of Public Health, Houston, Texas, United States of America; New York State Health Department and University at Albany, United States of America

## Abstract

**Background:**

Head and neck cancer (HNC) incidence, mortality and survival rates vary by sex and race, with men and African Americans disproportionately affected. Risk factors for HNC include tobacco and alcohol exposure, with a recent implication of human papillomavirus (HPV) in the pathogenesis of HNC. This study describes the epidemiology of HNC in the United States, examining variation of rates by age, sex, race/ethnicity and potential HPV-association.

**Methods:**

We used the North American Association of Central Cancer Registries (NAACCR) Cancer in North America (CINA) Deluxe Analytic Data to analyze HNC incidence for 1995–2005 from forty population-based cancer registries. We calculated age-adjusted incidence rates and incidence trends using annual percent change by age, sex, race/ethnicity and HPV-association.

**Results:**

Males and Non-Hispanic Blacks experienced greater HNC incidence compared to women and other race/ethnicity groupings. A significant overall increase in HNC incidence was observed among HPV-associated sites during 1995–2005, while non HPV-associated sites experienced a significant decline in HNC incidence. Overall, younger age groups, Non-Hispanic Whites and Hispanics experienced greater increases in incidence for HPV-associated sites, while HNC incidence declined for Non-Hispanic Blacks independent of HPV-association. In particular, for HPV-associated sites, HNC incidence for Non-Hispanic White males aged 45–54 increased at the greatest rate, with an APC of 6.28% (p<0.05). Among non HPV-associated sites, Non-Hispanic Black males aged 0–44 years experienced the greatest reduction in incidence (APC, −8.17%, p<0.05), while a greater decline among the older, 55–64 year age group (APC, −5.44%, p<0.05) occurred in females.

**Conclusions:**

This study provides evidence that HPV-associated tumors are disproportionately affecting certain age, sex and race/ethnicity groups, representing a different disease process for HPV-associated tumors compared to non HPV-associated tumors. Our study suggests that HPV tumor status should be incorporated into treatment decisions for HNC patients to improve prognosis and survival.

## Introduction

In the United States, it is expected that 36,540 new cases of oral, pharyngeal, and laryngeal cancer, also known as head and neck cancer (HNC), will be diagnosed in 2010, and 7,880 deaths will occur from HNC [1], [2]. The term head and neck cancer represents a broad range of anatomic sub-sites and often there is no consensus as to what locations should be included under this all-encompassing, yet generic label. However, customarily this term includes the following anatomic locations: lip, tongue, gum, floor of mouth, palate, oral cavity, salivary glands, tonsils, oropharynx, nasopharynx, hypopharynx, nasal cavity, paranasal sinus, and larynx.

HNC incidence, mortality, and relative survival rates vary with respect to sex and race. Incidence and mortality rates are more than twice as high in men as in women, and are greatest in men who are older than 50 years of age [2], [3]. African American men have higher incidence and mortality rates for HNC compared to White men. Incidence rates for African American and White women are fairly similar, although African American women have slightly higher mortality rates than White women [3], [4]. While there have been advances in treatment modalities, five-year relative survival has remained low (61%) and relatively unchanged for the past three decades [2], [4], [5]. Both incidence and mortality rates depend upon the anatomic location of the tumor and vary considerably. The tongue and oropharynx are the two anatomic sub-sites with the highest incidence and mortality [6]. In addition, tumor stage strongly influences survival. Localized tumors demonstrate an approximate 83% five-year relative survival; in contrast, regional tumors demonstrate a 54% five-year relative survival and distant tumors have a 32% five-year relative survival [2].

It is well known that intensity and duration of alcohol and tobacco consumption are established risk factors for HNC. Over 80% of HNC cases are directly attributable to alcohol and tobacco exposure, however, the other 15–20% of cases generally consist of non-smokers and non-drinkers [7]. This suggests that other risk factors besides alcohol and tobacco use are important in the etiology of HNC. Recent epidemiological and experimental data has implicated infection with human papillomavirus (HPV) in the pathogenesis of HNC [8]. It has been reported that approximately 25% of all HNC are positive for HPV-DNA, with 90–95% of those positive for HPV type 16 [8], [9], [10]. Applebaum, et al. [11] have reported HPV type 16 seropositivity is associated with an approximately four-fold increased risk of HNC. HPV detection has been observed to vary considerably across HNC sub-sites, with HPV prevalence significantly higher in the oropharynx and tonsil than the oral cavity and larynx [9], [10], [11], [12].

With the implication of HPV infection as an emergent risk factor for HNC, it is crucial to describe the distribution and incidence of HNC by sub-sites potentially associated with HPV and those not associated with HPV. The disparity of HNC among sex and race/ethnicity groups also needs better understanding to determine the potential causes for such large differences in incidence. This study uses 11 years of HNC incidence data (1995–2005) from the North American Association of Central Cancer Registries (NAACCR) Cancer in North America (CINA) Deluxe Analytic Data to investigate HNC trends in incidence by potential HPV-association with further stratification on age, sex and race/ethnicity. In comparison to SEER data, a greater geographic coverage and a larger sample size can be obtained with utilization of the CINA data, allowing for more in-depth analyses. This may yield patterns in HNC incidence that could not be seen in smaller sample sizes. The objectives of our study were to assess the incidence of HNC in the United States between 1995 and 2005 and to investigate incidence trends of HNCs putatively associated with HPV infection.

## Methods

Cases of HNC were identified through the NAACCR CINA Deluxe Analytic Data for the diagnosis years 1995 to 2005. The source of this data is from NAACCR data submissions as of December 2007. Support for cancer registries is provided by the state, province, or territory in which the registry is located. In the U.S., registries also participate in the National Cancer Institute's Surveillance, Epidemiology, and End Results (SEER) Program or the Centers for Disease Control and Prevention's National Program of Cancer Registries (NPCR) or both. To be included in the CINA Deluxe Analytic Data file, data from the central cancer registries must meet the NAACCR high quality standard (either gold or silver) on completeness of case ascertainment (90% or higher), passing edits (97% or greater), and other data quality indicators. All data from the registries were analyzed as aggregate data, and we excluded overlapping metropolitan areas to avoid duplication of case counts. The registries included in this data set are: Alabama, Alaska, Arizona, Arkansas, California, Colorado, Connecticut, Delaware, Florida, Georgia, Hawaii, Idaho, Illinois, Indiana, Iowa, Kentucky, Louisiana, Maine, Massachusetts, Michigan, Minnesota, Missouri, Montana, Nebraska, Nevada, New Hampshire, New Jersey, New Mexico, New York, North Dakota, Oklahoma, Oregon, Pennsylvania, Rhode Island, South Carolina, Texas, Utah, Virginia, Washington and Wyoming. To conduct the trend analyses, the data set was further restricted to only those registries that contributed data to CINA Deluxe for the complete 1995–2005 period. These registries are: Atlanta, California, Colorado, Connecticut, Delaware, Florida, Hawaii, Idaho, Illinois, Iowa, Kentucky, Louisiana, Maine, Michigan, Minnesota, Nebraska, New Jersey, New Mexico, New York, Pennsylvania, Rhode Island, Texas, Utah, Washington and Wyoming. Georgia's Cancer Registry did not contribute data during 1995–1998 and therefore, were excluded in the trend analysis. However, the City of Atlanta's Cancer Registry contributed data for the entire 1995–2005 time period, so this subset of Georgia's population was included in the trend analysis. The Louisiana State University Health Sciences Center's Institutional Review Board and NAACCR's Institutional Review Board reviewed and approved this study to be exempt. Consent did not need to be obtained since all data was de-identified and in an aggregate data form.

The CINA Deluxe dataset uses a joint race/ethnicity variable generated from race and ethnicity information found in medical records, which were then enhanced by the NAACCR Hispanic Identification Algorithm (NHIA) [13]. This joint race/ethnicity variable has five categories: Hispanic/All Races, Non-Hispanic/White, Non-Hispanic/Black, Non-Hispanic/Other and Non-Hispanic/Unknown. The Non-Hispanic/Other and Non-Hispanic/Unknown categories were excluded from the analysis since the racial/ethnic status of these HNC cases could not be obtained (7,077 cases, 2.6% of the total number of HNC cases).

Another important data issue occurs with the use of multiple staging systems during the eleven year time period of data collection. The stages of HNC diagnosed from 1995–2000 were classified according to the 1977 SEER summary staging system criteria, HNC diagnosed from 2001–2003 were classified according to the 2000 SEER summary staging system, and HNC diagnosed from 2004–2005 were classified according to the derived 2000 SEER summary staging system. The use of multiple staging systems can lead to potential misclassification and affect the stage distribution of the population.

To identify cases in the CINA deluxe analytic data file between 1995 and 2005 with HNC (including all anatomical sites under the heading of “lip, oral cavity, pharynx” and “larynx”) we extracted the following HNC sites according to the topography codes in the International Classification of Diseases for Oncology, Third Edition (ICD-O-3) [14]: tongue (C01.9–C02.9); gum (C03.0–C03.9); floor of mouth (C04.0–C04.9); palate (C05.0–C05.9); other and unspecified parts of the mouth (C06.0–C06.9); tonsil (C09.0–C09.9); oropharynx (C10.0–C10.9); hypopharynx (C12.9, C13.0–C13.9); other oral cavity and pharynx (C14.0, C14.2–C14.8); and larynx (C32.0–C32.9). HNC cases eligible for this analysis included incident, microscopically confirmed invasive cancers. The majority of oral and pharyngeal cancers reported are squamous cell in origin (95%), so we restricted the histology to include only those cancers of squamous cell origin (ICD-O-3 morphology codes: 8050–8052, 8070–8076, and 8081–8084). Three HNC sites, lip (C00.0–C00.9); salivary glands (C07.9–C08.9); and nasopharynx (C11.0–C11.9), were excluded in the analysis due to etiologic and histological differences from the other sub-sites.

We also classified all HNC cases into two groups based on proclivity for HPV infection: HPV-associated sites and non HPV-associated sites. Since we were unable to determine the true HPV status of the tumors we relied on professional expertise and available literature to determine which anatomic sub-sites are potentially associated with HPV [7], [9], [12], [15], [16], [17], [18], [19], [20], [21], [22], [23]. Three distinct anatomic sub-sites were classified as potentially associated with HPV. These three sites and their corresponding ICD-O-3 codes are: the tonsil, including the Waldeyer ring (C09.0–C09.9 and C142); the base of tongue and lingual tonsil (C01.9 and C02.4); and parts of the oropharynx (C10.2–C10.9). The remaining eligible HNC sites were included in the analysis as non HPV-associated sites.

### Statistical Analysis

Counts and percent distribution were computed by sex, age, race/ethnicity, and cancer stage for all HNC sites, as well as those sites associated with HPV infection and those sites not associated with HPV infection. HNC incidence counts, age-adjusted incidence rates (AAIR; number of new cases per 100,000 person-years standardized according to the US 2000 Standard Population) and corresponding 95% confidence intervals (95% CIs) were calculated by sex, race/ethnicity, cancer stage and proclivity for HPV infection. In addition, race/ethnicity specific rate ratios and corresponding 95% CIs were calculated using Non-Hispanic Whites as the referent group. Lastly, we calculated the annual percent change (APC) in incidence rates from 1995–2005 to assess whether incidence trends differ across time for HPV-associated and non HPV-associated sites as well as age, sex and race/ethnicity. All data analyses were conducted using the SEER*Stat software, Version 6.2.4 (National Cancer Institute, Bethesda, MD).

## Results

As shown in Table 1, 273,273 HNC cases were reported by the cancer registries included in the NAACCR CINA Deluxe Analytic Data file during 1995 to 2005. The majority of the cases were male (73.4%) and Non-Hispanic White (80.7%). Generally, HNC cases were more commonly diagnosed at older age groups, with the majority of cases diagnosed at 65 years of age or older (47.0%). HNC was diagnosed more frequently at a regional or localized stage (42.3% and 40.8% respectively), with 7.1% of the tumors diagnosed lacking staging information. Similar distributions were seen when cases were stratified into tumors potentially associated with HPV infection, with the majority of cases being of older age, male and Non-Hispanic White independent of HPV-association. Although overall the majority of cases were diagnosed at older ages, a difference in the age distribution is evident when stratifying by potential HPV-association. For Non-HPV associated sites, a little over half of the cases were diagnosed at 65 years or older (51.2%), whereas for HPV-associated sites about a third of the cases were diagnosed at the 55–64 year age group and the 65 and older age group (30.1% and 34.6% respectively). Diagnosis of regional stage tumors occurred more often among HNC cases associated with HPV, compared to the diagnosis of localized tumors occurring more frequently in non HPV-associated sites.

**Table 1:** Characteristics of study participants with incident HNC for HPV-Associated sites and non HPV-Associated sites by sex, age, race/ethnicity^a^ and stage^b^, 1995–2005.

		All HNC Sites		HPV-Associated Sites		Non HPV-Associated Sites	
Category	Sub-Category	Count	%	Count	%	Count	%
**Sex**	Male	200,497	73.4	53,391	77.5	147,106	72.0
	Female	72,776	26.6	15,470	22.5	57,306	28.0
**Age (Years)**	0–44	16,398	6.0	5,447	7.9	10,951	5.4
	45–54	52,587	19.2	18,874	22.4	33,713	16.5
	55–64	75,754	27.7	20,719	30.1	55,035	26.9
	65+	128,534	47.0	23,821	34.6	104,713	51.2
**Race/Ethnicity**	NH White	220,549	80.7	56,426	81.9	164,123	80.3
	NH Black	30,100	11.0	7,286	10.6	22,814	11.2
	Hispanic	15,547	5.7	3,650	5.3	11,897	5.8
**Stage**	Localized	111,604	40.8	11,086	16.1	100,518	49.2
	Regional	115,591	42.3	45,388	65.9	70,203	34.3
	Distant	26,739	9.8	8,268	12.0	18,471	9.0
	Unstaged	19,339	7.1	4,119	6.0	15,220	7.5
**All**		273,273	100	68,861	100	204,412	100

HNC: head and neck cancer; HPV: human papillomavirus; NH: Non-Hispanic.

a Any cases with an ‘Unknown’ or ‘Other’ race were not included in the analysis. Percentages for race/ethnicity will not total 100%.

b For 1995–2000, SEER Summary Stage 1997 was used; for 2001–2003, SEER Summary Stage 2000 was used; for 2004–2005, Derived SEER Summary Stage 2000 was used.

**Figure 1:**
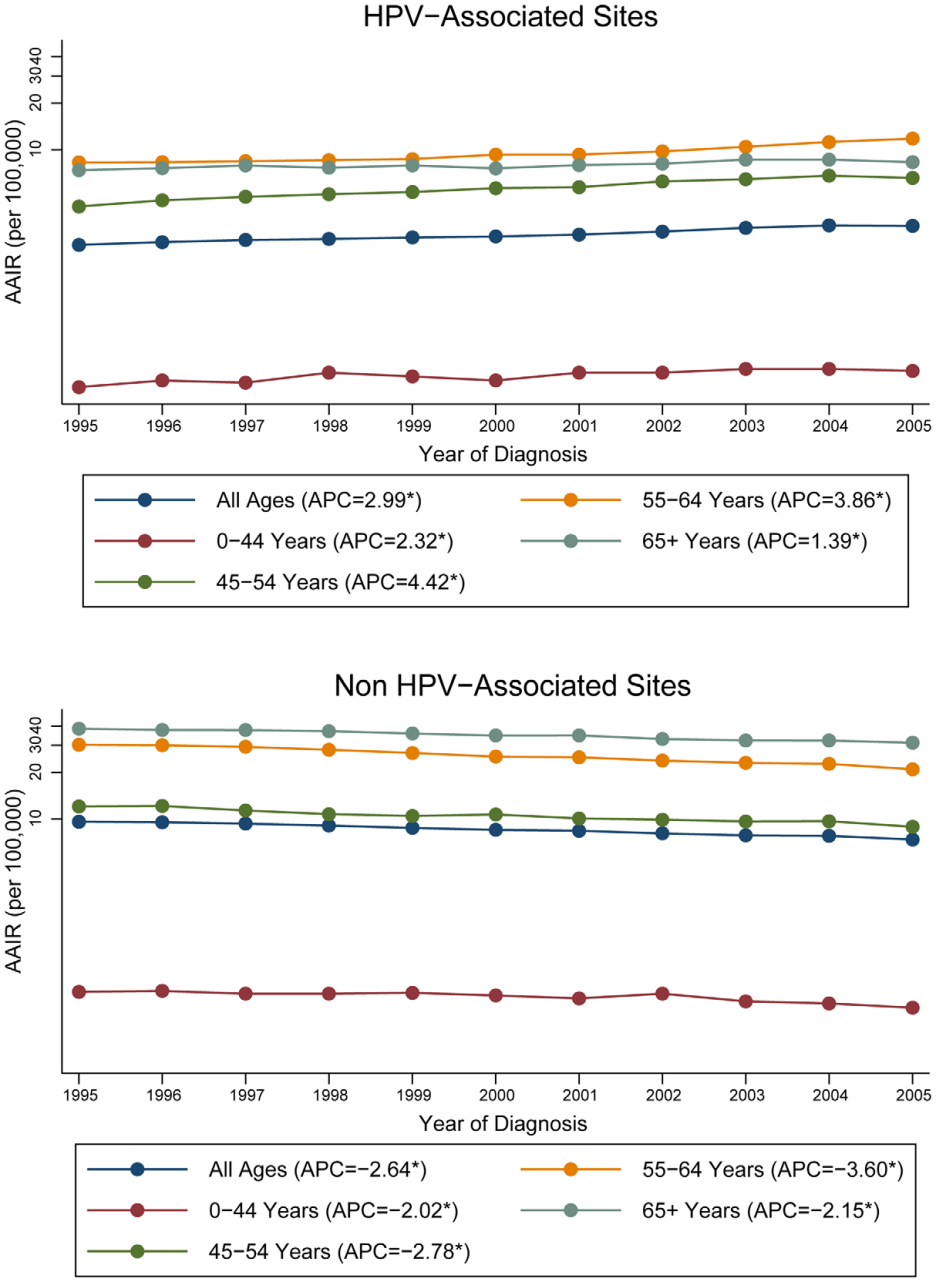
Annual percent change for age-adjusted HNC incidence rates by age, 1995–2005. AAIR: age-adjusted incidence rate; HNC: head and neck cancer; HPV: human papillomavirus; APC: annual percent change. *Rates are significantly different from zero (p<0.05). Rates are per 100,000 and age-adjusted to the 2000 US Standard Population (19 age groups – Census P25-1130). Annual percent change was calculated using the least squares method. Only registries contributing data to the entire 1995–2005 period were used in the analysis.

For HNC diagnosed in HPV-associated and non HPV-associated sub-sites, specific patterns emerged with respect to age (Table 2). Independent of HPV-association and sex, the 0–44 year age group experienced the lowest AAIR compared to the older age groups. Although the AAIRs for non HPV-associated sites are steadily increasing as age increases, a different pattern is seen in the AAIRs for the HPV-associated sites. Among the HNC diagnosed in HPV-associated sites, the AAIRs peak in the 55–64 year age group (9.73; 95% CI, 9.60–9.87) and decrease in the 65 and older age group. However, the AAIRs for females with HNC diagnosed in HPV-associated sites steadily increase with increasing age as seen for non HPV-associated sites. The same trends with respect to age can be seen over time by reviewing the APC in the AAIR for HPV-associated sites and non HPV-associated sites by age, shown in Figure 1. Over the eleven year time period, incidence rates increased over time irrespective of age for HPV-associated sites, while incidence rates decreased for non HPV-associated sites. In HPV-associated sites, the incidence for the 45–54 year age group increased at a greater rate than other age groups (APC, 4.42%, p<0.05). In general, for HPV-associated sites, the younger age groups experienced greater increases in incidence over the eleven year time period than the older age group. In non HPV-associated sites, the incidence for the 55–64 year age group decreased at a greater rate than the other age groups (APC, −3.60%, p<0.05).

**Table 2:** Age-adjusted incidence rates of potentially HPV-associated and non HPV-associated HNC for males and females by age and stage^a^, 1995–2005.

		Age-adjusted Incidence Rates (95% CI)					
		HPV-Associated Sites			Non HPV-Associated Sites		
Category	Sub-Category	Total	Male	Female	Total	Male	Female
**Age (Years)**	0–44	0.36 (0.35–0.37)	0.56 (0.55–0.58)	0.16 (0.15–0.17)	0.72 (0.70–0.73)	0.98 (0.96–1.00)	0.45 (0.44–0.47)
	45–54	5.91 (5.83–6.00)	10.14 (9.98–10.30)	1.85 (1.78–1.91)	10.57 (10.46–10.68)	16.52 (16.32–16.73)	4.84 (4.73–4.95)
	55–64	9.73 (9.60–9.87)	16.35 (16.10–16.60)	3.64 (3.52–3.75)	25.86 (25.64–26.07)	41.07 (40.68–41.47)	11.85 (11.65–12.06)
	65+	7.94 (7.84–8.04)	12.90 (12.71–13.10	4.21 (4.11–4.30)	34.91 (34.70–35.12)	57.15 (56.73–57.57)	18.71 (18.51–18.91)
**Stage**	Localized	0.46 (0.46–0.47)	0.71 (0.69–0.73)	0.25 (0.24–0.26)	4.21 (4.18–4.24)	6.84 (6.79–6.89)	2.10 (2.07–2.12)
	Regional	1.90 (1.88–1.92)	3.21 (3.17–3.24)	0.74 (0.72–0.75)	2.94 (2.92–2.96)	4.56 (4.52–4.60)	1.56 (1.53–1.58)
	Distant	0.35 (0.34–0.35)	0.59 (0.58–0.61)	0.13 (0.12–0.14)	0.77 (0.76–0.78)	1.27 (1.25–1.29)	0.36 (0.35–0.37)
	Unstaged	0.17 (0.17–0.18)	0.28 (0.27–0.29)	0.08 (0.07–0.08)	0.64 (0.63–0.65)	1.01 (0.99–1.03)	0.34 (0.33–0.35)
**All**		2.88 (2.86–2.91)	4.79 (4.75–4.83)	1.20 (1.18–1.22)	8.56 (8.52–8.60)	13.67 (13.60–13.74)	4.35 (4.31–4.38)

HNC: head and neck cancer; HPV: human papillomavirus; 95% CI: 95% Confidence Interval.

Rates are per 100,000 and age-adjusted to the 2000 U.S. Standard Population (19 age groups – Census P25-1130).

Confidence intervals (Tiwari model) are 95% for rates.

a For 1995–2000, SEER Summary Stage 1997 was used; for 2001–2003, SEER Summary Stage 2000 was used; for 2004–2005, Derived SEER Summary Stage 2000 was used.

Table 2 also outlines the differences in incidence by stage for HPV-associated sites and non HPV-associated sites. Across HPV-associated sites, regional staged tumors had the highest AAIR (1.90; 95% CI, 1.88–1.92), whereas for non HPV-associated sites, tumors diagnosed at a localized stage had higher AAIRs (4.21; 95% CI, 4.18–4.24). For both HPV-associated and non HPV-associated sites, tumors diagnosed at a distant stage had the lowest incidence when excluding unstaged tumors. The same patterns are evident when stage-specific incidence is stratified by sex.

Age-adjusted incidence rates of HNCs were highest among Non-Hispanic Blacks, followed by Non-Hispanic Whites and Hispanics (Table 3). The same patterns in race/ethnicity specific incidence are seen independent of sex and HPV-association. For all HNC sites, Non-Hispanic Blacks have a 25% increased risk of diagnosis with HNC compared to Non-Hispanic Whites (RR, 1.25; 95% CI, 1.24–1.27); while Hispanics have a 29% reduced risk of HNC diagnosis compared to Non-Hispanic Whites (RR, 0.71; 95% CI, 0.70–0.72). Non-Hispanic Blacks have significantly higher incidence rates compared to Non-Hispanic Whites except for females at all HNC sites and females diagnosed with HNC in non HPV-associated sites. The largest disparity between Non-Hispanic Blacks and Non-Hispanic Whites occurs in males diagnosed with HNC in a non HPV-associated site (RR, 1.50; 95% CI, 1.48–1.52). Hispanics have significantly lower incidence rates compared to Non-Hispanic Whites for all sex and potential HPV-association groupings. For Hispanics, the largest difference in incidence compared to Non-Hispanic Whites occurs among females, where there is about a 40% to 50% reduction in incidence depending on HPV-association.

**Table 3:** Age-adjusted incidence rates and rate ratios of potentially HPV-associated and non HPV-associated HNC by sex and race/ethnicity^a^, 1995–2005.

		Age-adjusted incidence rates (95% CI)			Rate ratios (95% CI)	
Category	Sub-Category	NH White	NH Black	Hispanic	NH Black/NH White	Hispanic/NH White
**All HNC Sites**	Total	11.71 (11.66–11.76)	14.67 (14.50–14.84)	8.34 (8.21–8.48)	1.25* (1.24–1.27)	0.71* (0.70–0.72)
	Males	18.56 (18.47–18.65)	26.45 (26.10–26.80)	14.61 (14.33–14.89)	1.43* (1.41–1.45)	0.79* (0.77–0.80)
	Females	5.84 (5.79–5.89)	5.96 (5.82–6.10)	3.34 (3.22–3.46)	1.02 (1.00–1.05)	0.57* (0.55–0.59)
**HPV-Associated Sites**	Total	3.04 (3.02–3.07)	3.45 (3.37–3.53)	1.83 (1.77–1.89)	1.13* (1.10–1.16)	0.60* (0.58–0.62)
	Males	5.00 (4.95–5.05)	6.12 (5.96–6.29)	3.25 (3.13–3.38)	1.22* (1.19–1.26)	0.65* (0.63–0.68)
	Females	1.27 (1.25–1.29)	1.39 (1.33–1.46)	0.64 (0.59–0.69)	1.10* (1.04–1.15)	0.51* (0.47–0.55)
**Non HPV-Associated Sites**	Total	8.67 (8.62–8.71)	11.22 (11.08–11.37)	6.51 (6.39–6.64)	1.30* (1.28–1.31)	0.75* (0.74–0.77)
	Males	13.56 (13.48–13.63)	20.33 (20.02–20.64)	11.36 (11.11–11.61)	1.50* (1.48–1.52)	0.84* (0.82–0.86)
	Females	4.57 (4.53–4.61)	4.57 (4.44–4.69)	2.69 (2.59–2.80)	1.00 (0.97–1.03)	0.59* (0.57–0.61)

HNC: head and neck cancer; HPV: human papillomavirus; 95% CI: 95% Confidence Interval; NH: Non-Hispanic.

Rates are per 100,000 and age-adjusted to the 2000 U.S. Standard Population (19 age groups – Census P25-1130).

Confidence intervals (Tiwari model) are 95% for rates and ratios.

* The rate is significantly different than the rate for Non-Hispanic Whites (p<0.05).

a Any cases with an ‘Unknown’ or ‘Other’ race were not included in the analysis.

**Figure 2:**
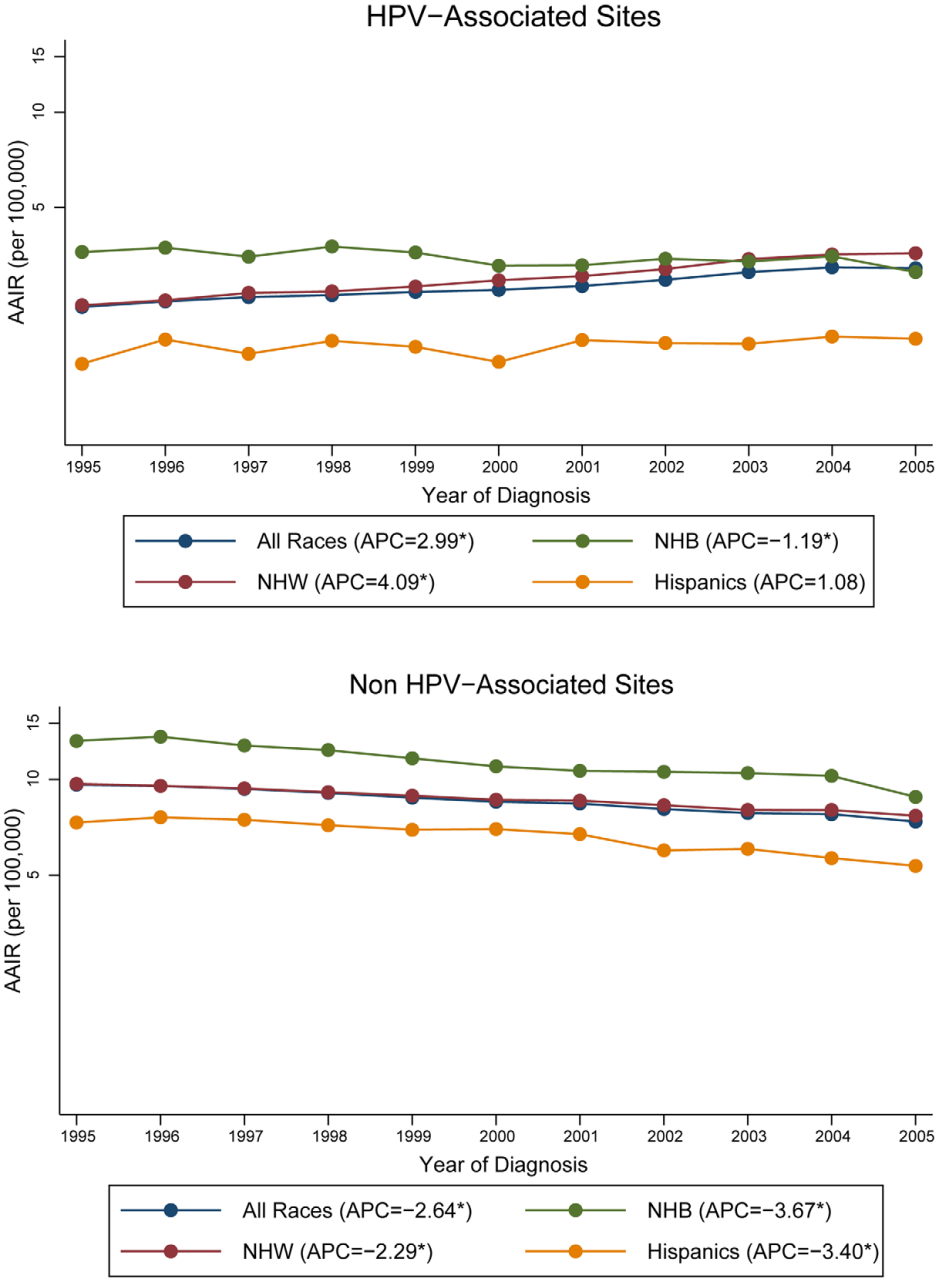
Annual percent change for age-adjusted HNC incidence rates by race, 1995–2005. AAIR: age-adjusted incidence rate; HNC: head and neck cancer; HPV: human papillomavirus; APC: annual percent change; NHW: Non-Hispanic Whites; NHB: Non-Hispanic Blacks. *Rates are significantly different from zero (p<0.05). Rates are per 100,000 and age-adjusted to the 2000 US Standard Population (19 age groups – Census P25-1130). Annual percent change was calculated using the least squares method. Only registries contributing data to the entire 1995–2005 period were used in the analysis.

Figure 2 illustrates the APC in the AAIR for HPV-associated sites and non HPV-associated sites by race/ethnicity for the years 1995 to 2005. A significant overall increase in incidence occurred in HNC diagnosed in a HPV associated sub-site, an APC of 2.99%; in contrast, we found a significant overall decrease in incidence during the same time period for the non-HPV associated sub-sites (APC, −2.64%, p<0.05). When stratified by race/ethnicity category, the increase in incidence among sub-sites associated with HPV is mainly seen among Non-Hispanic Whites (APC, 4.09%, p<0.05) and Hispanics (APC, 1.08%, p<0.05). In contrast, the incidence for Non-Hispanic Blacks decreased significantly at an APC of −1.19% over the same time period among sub-sites associated with HPV. Among non HPV-associated sites, HNC incidence significantly declined over the eleven year time period independent of race/ethnicity, with the largest decrease in incidence occurring among Non-Hispanic Blacks (APC, −3.67%, p<0.05).

**Figure 3:**
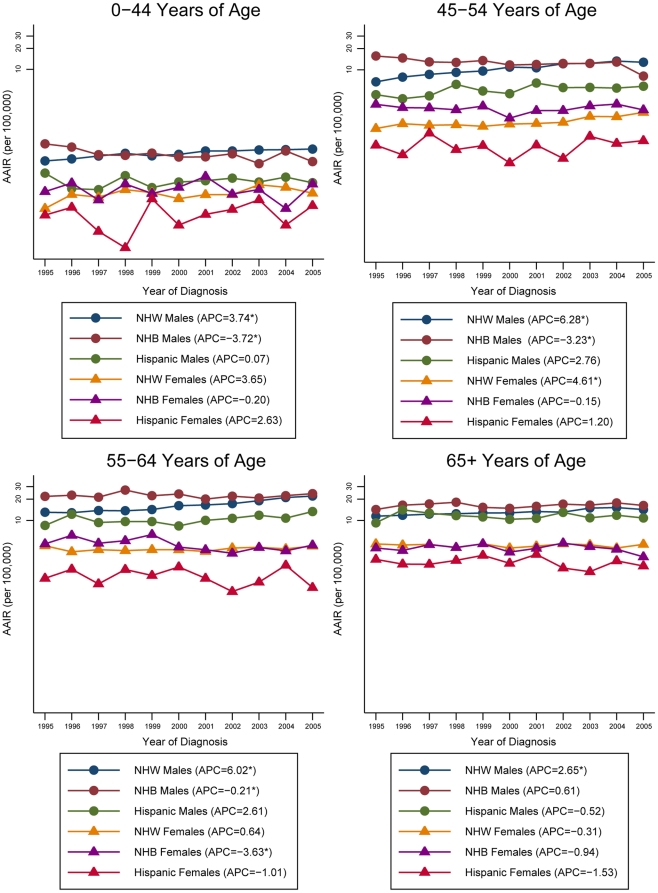
Annual percent change for age-adjusted HNC incidence rates by age, sex and race/ethnicity for HPV-associated sites, 1995–2005. AAIR: age-adjusted incidence rate; HNC: head and neck cancer; HPV: human papillomavirus; APC: annual percent change; NHW: Non-Hispanic Whites; NHB: Non-Hispanic Blacks. *Rates are significantly different from zero (p<0.05). Rates are per 100,000 and age-adjusted to the 2000 US Standard Population (19 age groups – Census P25-1130). Annual percent change was calculated using the least squares method. Only registries contributing data to the entire 1995–2005 period were used in the analysis.

**Figure 4:**
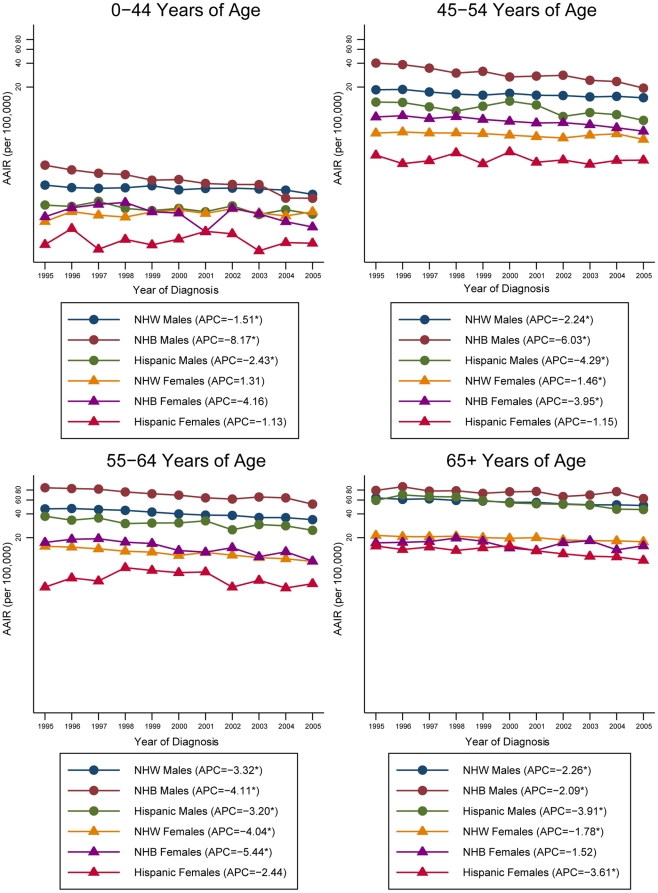
Annual percent change for age-adjusted HNC incidence rates by age, sex and race/ethnicity for Non HPV-associated sites, 1995–2005. AAIR: age-adjusted incidence rate; HNC: head and neck cancer; HPV: human papillomavirus; APC: annual percent change; NHW: Non-Hispanic Whites; NHB: Non-Hispanic Blacks. *Rates are significantly different from zero (p<0.05). Rates are per 100,000 and age-adjusted to the 2000 US Standard Population (19 age groups – Census P25-1130). Annual percent change was calculated using the least squares method. Only registries contributing data to the entire 1995–2005 period were used in the analysis.

Further investigation of the trends in the APC of the AAIR by HPV-association, age, sex and race/ethnicity can be found in Figures 3 and 4. For HPV-associated sites, younger Non-Hispanic Whites and Hispanics are experiencing the greatest increase in incidence in the HNC rates compared to the older age groups. In particular, the incidence rates for Non-Hispanic White males aged 45–54 years increased at the greatest rate, with an APC of 6.28% (p<0.05). For Non-Hispanic Blacks, declines in incidence occurred across all ages except for the older males (APC, 0.61%). Among Non-Hispanic Black males, the incidence rates declined more rapidly in the younger age groups compared to the older age groups. The Non-Hispanic Black females experienced the greatest decline in incidence in the 55–64 year age group (APC, −3.63%, p<0.05), while the incidence in the other age groups remained fairly constant. For non HPV-associated sites, declines in the incidence of HNC can be seen independent of age, race/ethnicity and sex. The steepest decline in incidence for Non-Hispanic Whites occurred in the 55–64 year age group independent of sex, with an APC of −3.32% (p<0.05) for males and an APC of −4.04% (p<0.05) for females. Young Non-Hispanic Black males experienced the greatest reduction in incidence (APC, −8.17%, p<0.05), while a greater decline among the older, 55–64 year age group (APC, −5.44%, p<0.05) occurred in females. A similar trend is evident for the Hispanics, with the greatest decline in incidence among younger males and older women.

## Discussion

Our analysis of HNC incidence in the United States reinforces previous observations that HNC incidence rates disproportionately affect men and Non-Hispanic Blacks [4], [17], [20], [24], [25], [26], [27]. We also provide evidence that infection with HPV has dramatically affected the disease distribution of HNC. In light of an overall decrease in HNC over the past two decades, HNC sub-sites most strongly associated with HPV have demonstrated a significant increase in incidence over time relative to non HPV-associated sites. One notable exception is the trend among Non-Hispanic Blacks, where the APC for both HPV-associated and non HPV-associated HNC sites demonstrates a downward trend over time. Similar to other studies, we also found rapid increases in HNC incidence among younger Non-Hispanic Whites and Hispanics in HPV-associated sites, while relatively large declines in HNC incidence occurred among non HPV-associated sites, particularly among young Non-Hispanic Blacks [22], [27], [28].

We found that a greater proportion of the subjects in this study with HNC in HPV-associated sites were diagnosed at younger ages compared to non HPV-associated sites. Gillison, et al. also reported a similar trend where HPV-positive patients were younger by about 5 years on average when compared to HPV-negative patients [29]. In a study by Ringstrom, et al., patients who were positive for HPV type 16 were 8.4 years younger than the HPV-negative group, and patients who were positive for HPV type 16 were more likely to be less than 59 years of age [19]. Other studies have shown similar findings with respect to age and HPV [18], [22]. There was also a similar pattern seen when HNC incidence was plotted over time by age, where overall, younger age groups had greater increasing trends in incidence for HPV-associated sites compared to older age groups. These findings suggest that HPV tumor status may be an important etiologic factor in the diagnosis of HNC in younger adults.

Subjects diagnosed with HNC in HPV-associated sites were more commonly diagnosed at a regional tumor stage compared to non HPV-associated sites, which were more commonly diagnosed at a localized stage. The same trend was seen among the stage-specific AAIRs. Other studies have found similar results, with HPV-related HNC cases diagnosed at more advanced stages than non HPV-related sites [18], [22]. Although HPV-positive HNC cases are diagnosed at later stages, a meta-analysis of survival has shown that HPV-positive HNC cases have a significantly improved overall survival and a lower recurrence risk than HPV-negative tumors [30]. Physicians should consider HPV infection and the extent of disease associated with these tumors to make treatment decisions based on each patient's tumor characteristics.

Since the 1980s, HNC incidence rates have been declining [24], [31], [32], [33]. In the current study, different trends were seen in HNC incidence rates once stratified by potential HPV-association. HPV-associated sites experienced an increase in incidence over the eleven year time period, while a decreasing trend was seen for non HPV-associated sites. Previous studies have also found similar results when classifying HNC sub-sites by potential HPV-association [17], [22], [27]. In a study on tonsillar cancer and HPV infection, investigators demonstrated an increase in incidence of tonsillar cancer and a parallel increase in HPV infection during the same time period [21]. Shiboski, et al. did not classify sub-sites based on HPV infection, but similar trends were seen with respect to incidence trends by sub-site: tongue and tonsil incidence rates have increased, whereas the incidence of other HNC sites has decreased or remained constant [34].

Several studies have found that patients with HPV-positive tumors are less likely to smoke and consume alcohol compared to patients with HPV-negative tumors [7], [10], [11], [18]. D'Souza, et al. found that exposure to HPV increased the association with oropharyngeal cancer regardless of tobacco and alcohol use [35]. This evidence suggests that the increase in HNC incidence among HPV-associated sites may be caused by other exposures besides tobacco and alcohol use. One such exposure that has been linked to HPV infection in the literature is high-risk sexual behavior, particularly oral-sex [10], [12], [18], [29], [35], [36], [37]. A similar association was also found in HIV positive individuals, suggesting that HPV can be transmitted in the oral cavity through sexual contact, particularly oral-genital contact [38], [39]. Patients engaging in high-risk oral sex behaviors are more readily exposed to HPV, which could lead to an increase in HNC incidence among HPV-associated HNC sub-sites.

Stratifying incidence rates over time by race/ethnicity revealed interesting patterns among certain race/ethnicity groupings. Among HPV-associated sites, the increasing trend in HNC incidence was mainly due to Non-Hispanic Whites. Interestingly, HNC incidence rates for Non-Hispanic Blacks are decreasing across HPV-associated sites as well as non HPV-associated sites. It was shown that in a study of HIV positive individuals, Caucasians had a greater risk of oral HPV infection than African Americans [40]. These findings suggest that the disparity in HNC incidence trends may be due to the disproportionate sexual behaviors among different race/ethnicities. Furthermore, in a study on the sexual behavior of men, Billy, et al. found that black men were less likely to have performed and received oral sex than white men [41]. If blacks are not engaging in high-risk sexual practices for oral HPV infection as suggested by Billy, et al. [41], the disparity in HNC incidence trends among HPV-associated sites for race/ethnicity seen in this paper can be explained. Future studies should examine the impact of sexual practices on HPV infection and the differences in sexual practices by race to determine the effect on HNC incidence rates.

There were several limitations to the current study. Due to the nature of the CINA data, we were unable to obtain individual risk factor and HPV level data on each HNC case. Inferences cannot be made as to individual level characteristics, but this study provides important information from a population perspective. The second limitation, and the most notable, is the classification of anatomic sub-sites as HPV-associated and non HPV-associated. We were unable to determine the HPV status of each tumor, and relied on previous literature to determine which anatomic sub-sites were potentially associated with HPV. The classification of HPV-association could result in misclassification; however without HPV typing results on each individual, the classification of HPV tumor status can only be estimated by previous etiologic knowledge presented in the literature. Another limitation results from unavailable data on tobacco or alcohol use within the cancer registries. Without this knowledge, we cannot establish the effect of tobacco and alcohol use on HNC incidence and infection with HPV.

This study has shown that HNC cases associated with HPV infection represent a different disease process and mechanism compared to HNC cases caused by alcohol and tobacco use. The findings of this study suggest that determination of HPV tumor status could be important in the decision of treatment therapies for patients. With the identification of HPV-tumor status, treatment could be tailored to the individual patient to improve prognosis and survival. In addition, the current treatment options could be improved by development of vaccine-mediated therapy or the use of biological modifiers for preservation therapy of organs within the head and neck region.
